# Identification and validation of *TUBB*, *CLTA*, and *FBXL5* as potential diagnostic markers of postmenopausal osteoporosis

**DOI:** 10.17305/bb.2025.12019

**Published:** 2025-08-11

**Authors:** Yue Tan, Yujing Wang, Qin Zhu, Yan Xue, Xuhao Ji, Zhenkun Li, Jiawen Shen, Chengming Sun, Shiqi Ren, Chenlin Zhang, Jianfeng Chen

**Affiliations:** 1 Research Center of Clinical Medicine, Affiliated Hospital of Nantong University, Medical School of Nantong University, Nantong, China; 2 Department of Hand Surgery, Affiliated Hospital of Nantong University, Medical School of Nantong University, Nantong, China; 3 Department of Rehabilitation, Affiliated Hospital of Nantong University, Medical School of Nantong University, Nantong, China; 4 Department of Orthopedics, The 3rd People’s Hospital of Dalian Affiliated to Dalian Medical University, Dalian, China; 5 Department of Orthopaedics, Qidong Hospital of Chinese Medicine, Nantong, China; 6 Department of Spinal Surgery, Jiangsu CM Clinical Innovation Center of Degenerative Bone & Joint Disease, Wuxi TCM Hospital Affiliated to Nanjing University of Chinese Medicine, Wuxi, China

**Keywords:** m6A, postmenopausal osteoporosis, PMOP, m6A methylation, diagnostic biomarkers, unsupervised clustering

## Abstract

Postmenopausal osteoporosis (PMOP) is recognized as the most prevalent bone disease worldwide. N6-methyladenosine (m6A) is one of the most common RNA modifications influencing the progression of various disorders; however, its specific role in PMOP remains unexplored. This study aims to investigate the expression profiles of m6A-related genes and their impact on the prognosis of PMOP patients. We utilized the GSE56815 expression analysis dataset obtained from the Gene Expression Omnibus (GEO) database and extracted m6A-related genes for further examination. Our analysis revealed that m6A-related genes exhibited differential expression between PMOP patients and healthy controls. We employed consensus clustering to identify subgroups within the PMOP cohort and conducted immunological analyses on these clusters. Additionally, we intersected the clusters to identify differentially expressed genes (DEGs) and analyzed potential diagnostic markers for PMOP using support vector machine recursive feature elimination (SVM-RFE), LASSO, and random forest (RF) algorithms, which were subsequently validated in the GSE56116 dataset. The receiver operating characteristic (ROC) curve was employed to assess the diagnostic significance of these markers. Furthermore, quantitative PCR (qPCR) was performed to validate the expression of the identified genes. In the GSE56815 dataset, we identified three subtypes associated with m6A modifications, leading to the identification of 302 shared DEGs among these subtypes. Gene ontology (GO) analysis indicated that the DEGs were predominantly enriched in nuclear specks, the nuclear envelope, and nucleocytoplasmic transport processes. Kyoto Encyclopedia of Genes and Genomes (KEGG) enrichment analysis further revealed that DEGs were primarily associated with endocytosis and nucleocytoplasmic transport pathways. Through the application of SVM, LASSO, and RF algorithms, we identified three potential diagnostic markers: *TUBB*, *CLTA*, and *FBXL5*, which demonstrated promising diagnostic capabilities when tested against an independent dataset. qPCR validation confirmed significant expression differences of these genes between the control and PMOP groups. The genetic markers identified in this study hold potential for accurately predicting the risk of PMOP in patients. The findings contribute to understanding the underlying molecular mechanisms of *CLTA*, *TUBB*, and *FBXL5* in PMOP and may facilitate the development of novel therapeutic strategies and improved monitoring of the disease.

## Introduction

Postmenopausal osteoporosis (PMOP) is a prevalent metabolic bone disease that primarily affects postmenopausal women. Due to decreasing estrogen levels and increased bone resorption, menopause causes the destruction of bone microstructure and a reduction in bone mass, as well as increased brittleness [1]. Secondary osteoporotic fractures raise the social burden by increasing disability and mortality, especially psychologically and economically. It is a challenge for the healthcare system to address osteoporosis due to its various complications and the high personal and social costs, particularly as the majority of affected individuals do not receive treatment. This lack of health initiatives is evident in nearly 60% of affected individuals who are at high risk for osteoporotic fractures and do not receive bone protective therapy [2]. Currently, PMOP treatment primarily relies on bisphosphonates, selective estrogen receptor modulators (SERMs), calcitonin, RANKL inhibitors, parathyroid hormone analogues, and Wnt pathway agonists [3]. Nevertheless, these therapies still face obstacles regarding adherence, safety, and personalized efficacy, while current diagnostic approaches (e.g., DXA) have poor sensitivity and cannot accurately predict fracture risk. Thus, it is necessary to identify more precise molecular markers for the early diagnosis and personalized treatment of PMOP.

The most prevalent RNA methylation is N6-methyladenine (m6A) [4], which occurs in the conserved RRACH motif (R ═ G or A; H ═ A, C, or U) [5] and is concentrated close to the stop codon of mRNAs [6]. Notably, m6A modification enzymes have been identified as enzyme technology has progressed. The major m6A methyltransferases are the methyltransferase-like protein, methyltransferase, and Wilms tumor-associated protein complex, while fat and obesity-related protein (FTO) and α-ketoglutarate-dependent [7, 8] dioxygenase base B homolog (ALKBH5) are the main demethylases. Presently, among RNA modifications, m6A has become a research hotspot [9]. The aforementioned modification performs various regulatory roles in post-transcription, such as transcriptional regulation, alternative splicing, stabilization, and translation, by binding to YTH domain-containing proteins [10]. Additionally, m6A modification regulates cellular biological functions, including cell differentiation, embryo development, and disease incidence. Bones perform a vital role in providing support and protection to the body; although these are just a few of the many other functions performed, including enabling movement, blood production, and acting as a reservoir for various minerals such as calcium. A variety of epigenetic modifications influence the expression of genes in bone cells through various regulatory mechanisms, thereby affecting the precise processes of remodeling and development of bones. These mechanisms include histone modification, along with DNA and RNA methylation [11]. Prior research has indicated a significant effect on the activity and function of bone cells due to the disruption of epigenetic processes, which could potentially lead to the development of bone-related disorders [10, 11].

Based on the Integrated Gene Expression Omnibus (GEO) database, consensus clustering was used to determine the clustering of m6A-DEGs, and the Least Absolute Contraction and Selection Operator (LASSO) algorithm [12], support vector machine-recursive feature elimination (SVM-RFE) algorithm [13], and random forest (RF) algorithm [14] were used to determine the diagnostic markers. The three most representative genes, *TUBB*, *CLTA,* and *FBXL5*, were verified in GSE56116 [15]. Moreover, we explored the probable molecular underpinnings of osteoporosis, ultimately benefiting the disease’s early detection, therapy, and prevention.

## Materials and methods

### Data retrieval

GEO datasets were utilized to acquire patient information (GSE56815) [16]. GSE56815 contains 80 samples, including 40 postmenopausal osteoporosis samples and 40 premenopausal samples. The m6A regulators that were removed included nine writers, 15 readers, and two erasers.

### m6A-DEGs in PMOP

The GSE56815 dataset underwent stringent data pre-processing operations to achieve comparability and reproducibility in this study. First, the raw expression data were processed by quantile normalization and log2 conversion to remove technical noise and optimize the data distribution. Second, we corrected the data with ComBat (sva R package) to eliminate batch effects and checked for batch effects with PCA to enable comparability between different datasets. Then, the limma (v3.58.1) software package was used to screen differentially expressed genes (DEGs), with a screening criterion of a Benjamini–Hochberg false discovery rate (FDR) adjusted *P* value of less than 0.05. The specific markers *TUBB*, *CLTA*, and *FBXL5* were validated in other datasets via cross-validation to provide robustness and reliability.

### Construction of m6A clusters based on m6A-associated genes

Various m6A clusters were built considering the expression of m6A-associated genes, while unsupervised clustering methods were performed to categorize patients into discrete classifications. The use of the consensus clustering technique ensured the quantity and stability of the clustering process, and the “ConsensusClusterPlus” (v1.66.0) software was used to assess the classification’s stability.

### Inference of microenvironment and immune cells

The immune cell infiltration characteristics of the PMOP group and control group were evaluated through single-sample gene set enrichment analysis (ssGSEA). Reference was made to the MSigDB and xCell immune cell marker gene sets, which included 24 types of immune cells, and the ssGSEA score was calculated using the GSVA (v1.50.5) R package. We used Z-score normalization to compare the differences in each observation within samples and Wilcoxon rank-sum tests for the assessment of group differences, and we performed Spearman correlation analysis to evaluate the correlation between core genes and immune cells. The results dissected the immune microenvironment in the PMOP group and investigated the potential function of *TUBB*, *CLTA*, and *FBXL5* in immune regulation.

### Identification of m6A gene expression between different patterns

In order to better study the pathway of enrichment in degree, we chose three intersecting genes, A-B, B-C, and A-C, for Kyoto Encyclopedia of Genes and Genomes (KEGG) pathway analysis and gene ontology (GO) biological method using a *P* value less than 0.05, a minimum count of 5, and an enrichment factor larger than 0.15. By installing the clusterProfiler (v4.10.1) R package, gene set enrichment analysis (GSEA) was utilized to analyze the subtype-associated activities of all genes considering their log2 fold change.

**Table 1:** Primer sequences for target genes

**Gene names**	**Forward primer**	**Reverse primer**
*TUBB*	TCCATGAAGGAGGTCGATGA	CAGACGGCTGTCTTGACATT
*CLTA*	CCGCCATGGCTGAGTTAGA	GGCTCTTCAGTGCACCAG
*FBXL5*	GGCAGATTTTAGAGCTTTGTCCTA	CGAAGACTCTGGCAGCAACCAA
*GAPDH*	GGAGTCCACTGGTGTCTTCA	GGGAACTGAGCAATTGGTGG

### Acquisition of diagnostic markers

A detailed study of the efficacy of the major biomarkers was conducted through algorithm screening. Three types of intersection genes, A-B, B-C, and C-A, were selected for algorithm screening, including minimum absolute contraction and selection operator (LASSO) logistic regression, SVM-RFE, and RandomForest (RF) [14, 17, 18]. These algorithms were utilized to identify the relevant PMOP-associated genes. The precise and efficient diagnostic biomarkers were detected through the intersection of the screened PMOP-related feature genes, and further analysis was performed.

### Screening and verification of diagnostic markers

The degree of accuracy regarding the predictive capability of the aforementioned diagnostic markers for patients with varying risk outcomes was examined by deriving the area under the curve (AUC) of the receiver operating characteristics (ROC), where an increased AUC value denotes an increased accuracy of the constructed gene signature. The box diagram of expression differences of core genes was drawn to examine the variation in the expression of the diagnostic markers between postmenopausal and premenopausal samples. We then obtained the gene for further verification in another dataset (GSE56116). The PerformanceAnalytics R package was employed to conduct a heat map analysis of gene correlation for the hub genes to analyze whether there is a correlation between the diagnostic markers and whether a correlation exists between their expression levels. The number cex = 0.7 was considered to be the correlation coefficient.

### To construct a nomogram model of postmenopausal osteoporosis and premenopausal classification

The “rms” (v6.8-1) software creates the nomogram model with consideration of the chosen explanatory variables. Based on the qualities of each variable for the patient, we project upwards on a tiny scale to determine the value of each item (point). The sum is derived from each item’s score. The greater the overall value, the greater the PMOP probability. The curves for calibration, decision curve analysis (DCA), and clinical effect were analyzed to confirm the model’s correctness. A 1000-resampling method was used for internal validation of the model.

### Single sample gene set enrichment analysis and correlation heat map of hub genes

Single sample gene set enrichment analysis (GSEA) was executed on the diagnostic markers screened through LASSO, SVM, and RF to analyze the corresponding functions of high and low expression of each hub gene. KEGG analysis was performed, and the first five and the last five GSEA results were selected and displayed together after ranking the analysis results.

### Quantitative real-time polymerase chain reaction (qRT-PCR)

This study collected ten peripheral blood samples, including five samples from healthy patients and five samples from PMOP. This study has been approved by the Medical Ethics Committee of Nantong University Affiliated Hospital. Samples were collected and used according to approved guidelines. Total RNA was extracted using the TRIzol method. Quantitative reverse transcription-polymerase chain reaction (qRT-PCR) was performed on RNA (2 µg) extracted from each sample on a LightCycler 480 PCR system (Roche, USA) using FastStart Universal SYBR Green Master. cDNA was used as a template in a 20 µL reaction volume (2 µL cDNA template, 10 µL PCR mixture, 0.5 µL forward and reverse primers, and an appropriate amount of water). The PCR reaction was performed using the following procedure: cycling conditions started with an initial DNA denaturation phase at 95^∘^C for 30 s, followed by 45 cycles at 94^∘^C for 15 s, 56^∘^C for 30 s, and 72^∘^C for 20 s. Three separate analyses were performed for each sample. Threshold cycle (CT) data were obtained based on the 2^-ΔΔCT^ method and normalized to GAPDH levels for each sample. It was confirmed that the melting curve was checked to ensure single amplicon specificity. The sequence list of primer pairs for target genes is shown in Table 1.

### Statistical analysis

The relationships between the genes (part of the gene signature) and the immune-associated cells were analyzed, and the characteristic gene expression in the case group was compared with that of the normal group for a more detailed investigation. The former correlation was determined using Spearman’s rank correlation coefficient, whereas the latter process was evaluated through the Wilcoxon signed-rank test. The AUC of the ROC curve was measured using the timeROC (v 0.4) package to examine the accuracy of the predictive ability of genes associated with PMOP for the probability of belonging to the PMOP group. The prediction ability was associated with the AUC values, wherein the general predictive value was set in the range of AUC > 0.60, while a value of AUC > 0.70 was set as a good predictive value. The R-4.0.3 software was utilized for statistical analysis in this research. A *P* value < 0.05 was considered statistically significant.

## Results

### m6A-DEGs in PMOP

**Figure 1.**
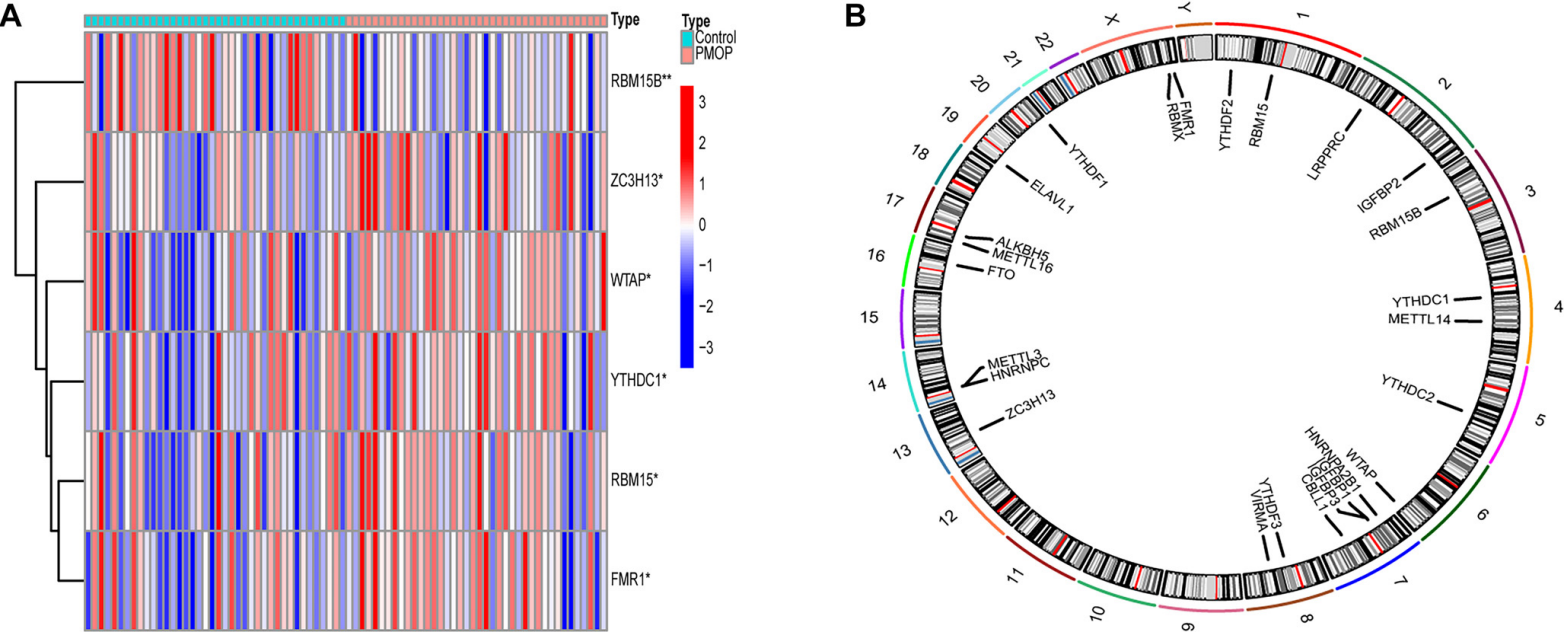
**The expression levels of m6A RNA methylation regulators between postmenopausal and premenopausal samples.** (A) Heatmap of m6A RNA methylation regulator expression levels in each sample; (B) The chromosomal positions of the m6A-DEGs. *P < 0.05; **P < 0.01; ***P < 0.001.

Firstly, six m6A-related DEGs were identified between the control groups and PMOP groups. Further, we drew the expression heatmap of six m6A DEGs (Figure 1A) and found that the expressions of WTAP, ZC3H13, RBM15, YTHDC1, and FMR1 in the PMOP group were higher than those in the control group (*P* < 0.01). In the control group, RBM15B expression was greater than that in the PMOP group. The chromosomal positions of the m6A-DEGs were plotted on a loop graph (Figure 1B).

### Consensus clustering of m6A genes among three clusters

**Figure 2.**
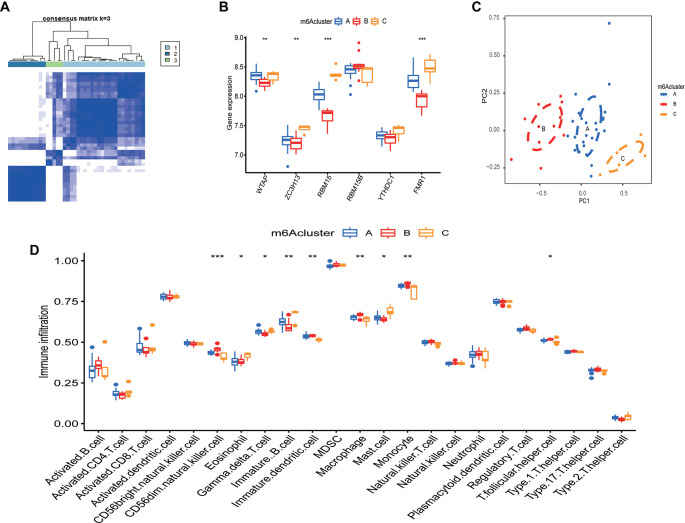
**Biological characteristics of distinct m6A clusters.** (A) Consensus clustering matrix for *k* ═ 3; (B) A boxplot of the genes clustered in these three m6A clusters; (C) Sample clustering by PCA; (D) The results of the GSEA scores in PMOP. *P < 0.05; **P < 0.01; ***P < 0.001.

The m6A-related genes were categorized according to their varying expression levels into different clusters. The crossover among PMOP samples was detected to be minimal when the consensus matrix K value was at 3 (Figure 2A). The expression of six m6A regulators in the clusters was studied, and the associated boxplots were plotted (Figure 2B). The resulting plots depicted the increased expression of WTAP in Cluster A compared to that in other clusters (*P* < 0.01). The expression level of RBM15B in Cluster B was elevated compared to the other clusters (*P* < 0.01), while the expressions of ZC3H13, RBM15, YTHDC1, and FMR1 in Cluster C were at a higher level than those in other clusters (*P* < 0.01). Additionally, the three distinct patterns of distribution of PCA lend credence (Figure 2C) to the classification generated by consensus clustering analysis. Increasing data show that osteoporosis advancement is strongly associated with the immune milieu, leading to the notion that the immune microenvironment can play a role in postmenopausal osteoporosis progression. Therefore, the variation in the infiltration status of lymphocytes was investigated. As shown in the figure (Figure 2D), immune cell infiltration landscapes across the three groups were significantly different. Immune cell infiltration analysis of the bone microenvironment showed that the m6A B cluster was significantly correlated with CD56 dim cells, immature dendritic cells, macrophages, and monocytes in peripheral blood. m6A C was significantly associated with immature B cell monocytes.

### Immune microenvironment of the three clusters

**Figure 3.**
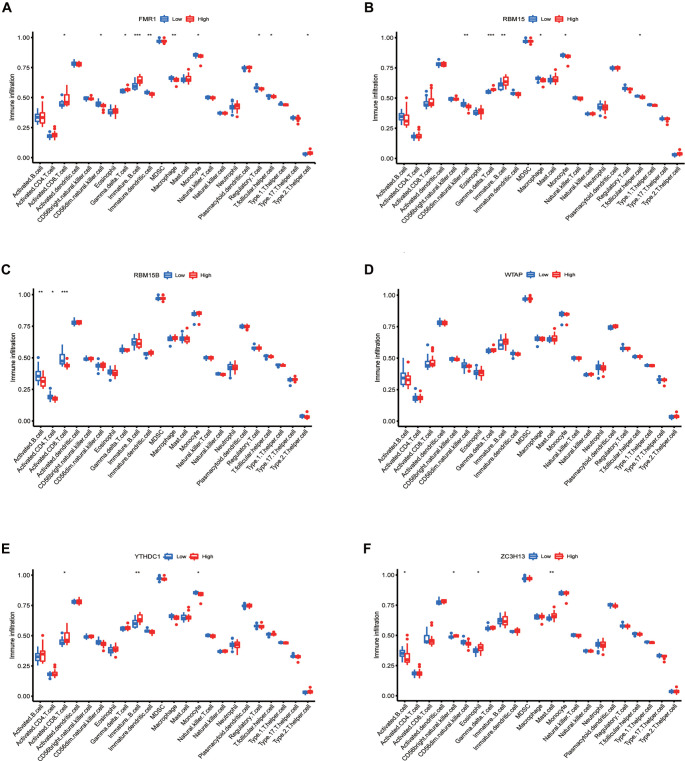
**Expression of genes in immune cells in PMOP.** (A) The expression of FMR1; (B) The expression of RBM15; (C) The expression of RBM15B; (D) The expression of WTAP; (E) The expression of YTHDC1; (F) The expression of ZC3H13. *P < 0.05; **P < 0.01; ***P < 0.001.

The immune microenvironment analysis of the six m6A regulators showed that the expression of FMR1 is high in immature B cells but low in immature dendritic cells and macrophages (Figure 3A). RBM15 was expressed at a high level in gamma delta T cells and immature B cells, while it was expressed at a low level in CD56dim natural killer cells (Figure 3B). RBM15B was underexpressed in activated CD8 T cells and activated B cells (Figure 3C). The expression of WTAP in the immunological microenvironment was not substantially different (Figure 3D). The immature YTHDC1 has enhanced expression in immature B cells (Figure 3E). ZC3H13 is highly expressed in mast cells (Figure 3F).

### DEGs identification and functional annotation

**Figure 4.**
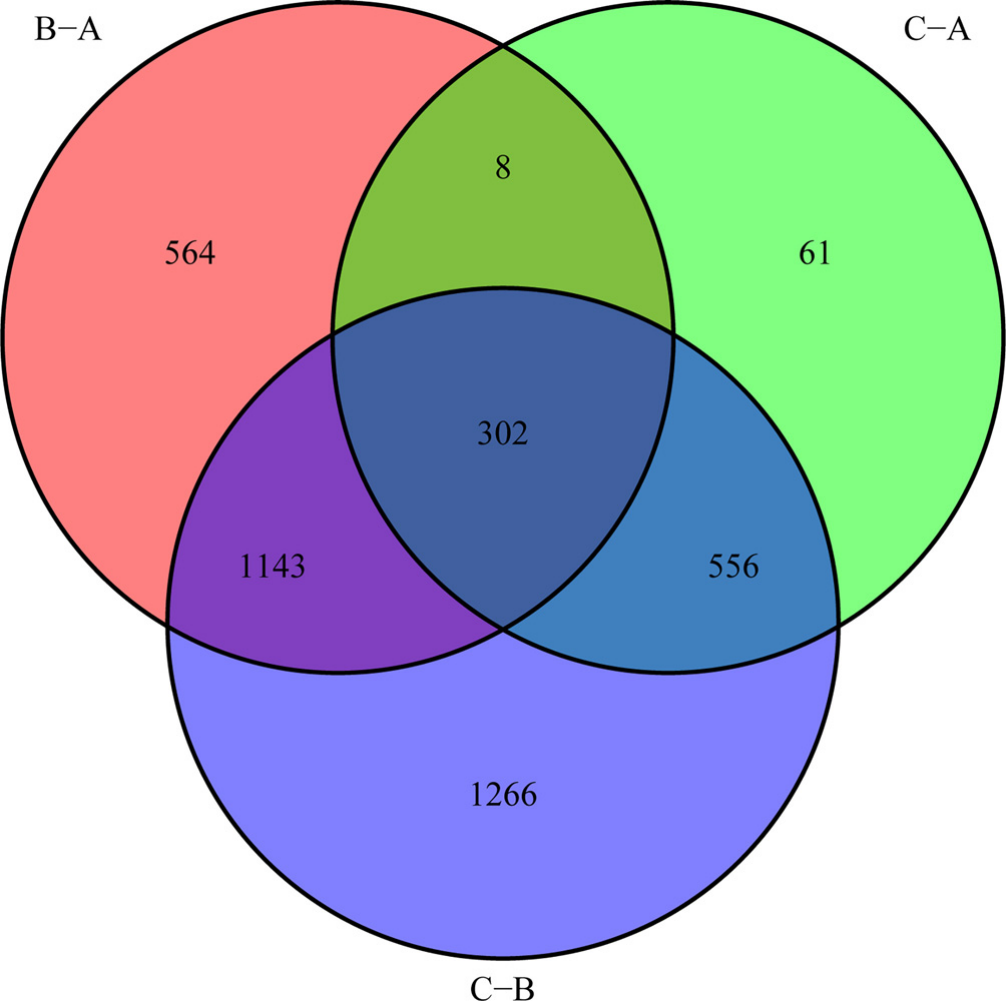
**Venn diagram of DEGs.** There were 302 DEGs among the three m6A gene patterns.

**Figure 5.**
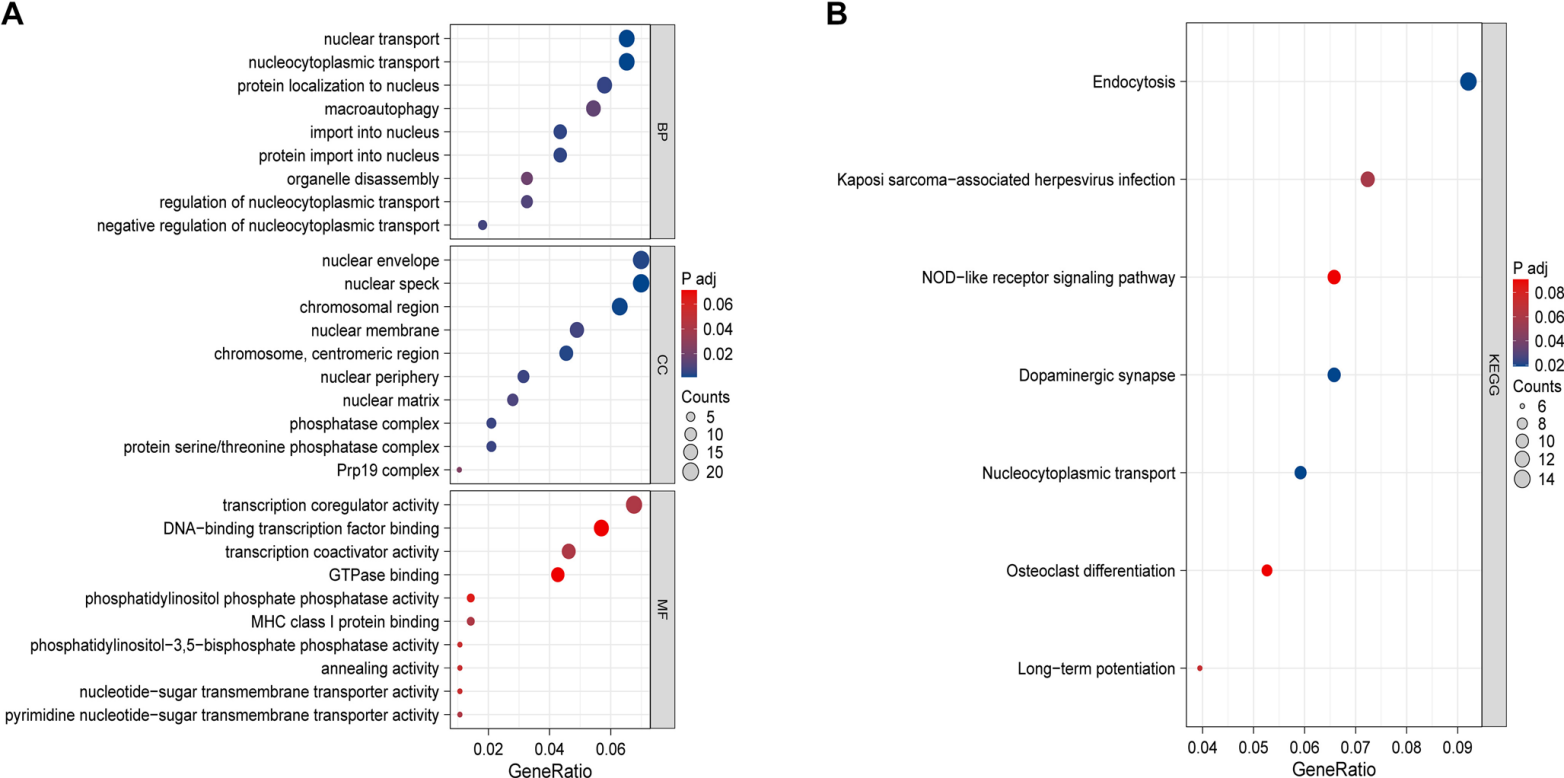
The differentially expressed genes were analyzed using GO and KEGG.

To explore the potential biological differences of the m6A cluster, we obtained 302 shared genes among three subgroups: Cluster A, Cluster B, and Cluster C (Figure 4). In addition, we performed GO and KEGG analyses on the 302 DEGs. The GO analysis of the biological mechanisms depicted enrichment of intersection genes in nuclear transport, protein localization to the nucleus, and nucleocytoplasmic transport. The cytological analysis of the aforementioned genes depicted their abundance in nuclear specks and the nuclear envelope, whereas their increased presence in transcription coregulator activity was exhibited through analyzing molecular biological functions (Figure 5A). The KEGG analysis of the biological mechanisms depicted nucleocytoplasmic transport (Figure 5B).

**Figure 6.**
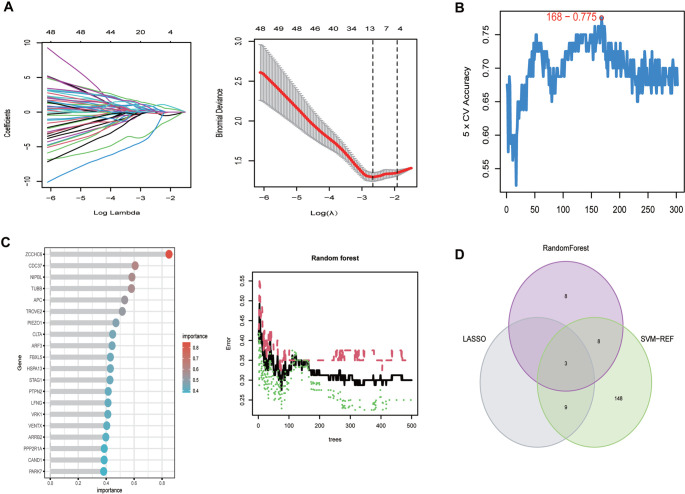
**The selection of postmenopausal osteoporosis (PMOP) feature genes by three algorithms.** (A) Least absolute contraction and selection operator (LASSO) algorithm; (B) Support vector machine-recursive feature elimination (SVM-RFE) algorithm; (C) Random forest (RF) algorithm; (D) The intersection of characteristic genes screened by the three algorithms.

### Screening and verification of diagnostic markers

**Figure 7.**
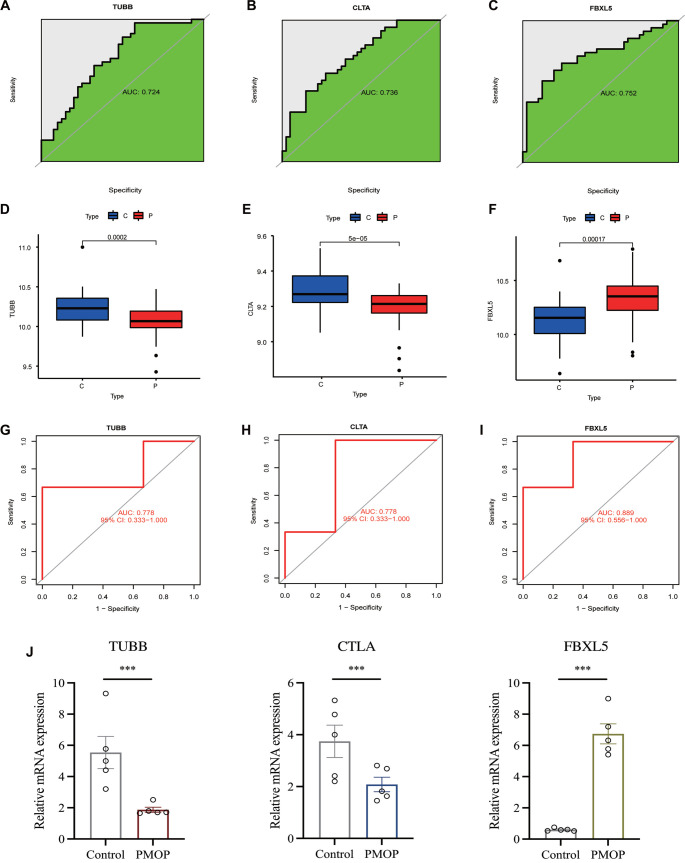
**Screening and verification of diagnostic markers.** (A) The AUC value of *TUBB* was 0.724; (B) The AUC value of *CLTA* was 0.736; (C) The AUC value of *FBXL5* was 0.752; (D) The expression of *TUBB* in the normal groups was higher than that in the PMOP groups; (E) The expression of *CLTA* in the normal groups was higher than that in the PMOP groups; (F) The expression of *FBXL5* in the PMOP groups was higher than that in the normal groups; (G) The AUC value of *TUBB* was 0.778; (H) The AUC value of *CLTA* was 0.778; (I) The AUC value of *FBXL5* was 0.889; (J) Differential expression of *TUBB*, *CLTA*, and *FBXL5* between the control group and PMOP group. *P < 0.05; **P < 0.01; ***P < 0.001. C: Control; P: PMOP.

We utilized the LASSO algorithm to identify the major 12 biomarkers out of a total of 302 cross-genes (Figure 6A). The major biomarkers from the cross-genes were detected by means of the SVM-RFE algorithm and were found to be 168 genes (Figure 6B). The RF algorithm was used to screen 19 key biomarkers from 302 cross-genes (Figure 6C). After that, we intersected the genes obtained by the three algorithms to obtain the three diagnostic markers *TUBB*, *CLTA*, and *FBXL5* (Figure 6D). The ROC curve of *TUBB*, *CLTA*, and *FBXL5* showed that they could be used as valuable biomarkers. The AUC value of *TUBB* was 0.724 (Figure 7A), that of *CLTA* was 0.736 (Figure 7B), and that of *FBXL5* was 0.752 (Figure 7C), indicating that these three diagnostic markers had high prediction accuracy. Differential expression analysis of these three genes exhibited that the levels of expression of *TUBB* (Figure 7D) and *CLTA* (Figure 7E) in the samples of normal patients were higher than those of PMOP patients, whereas the results were vice versa for *FBXL5* (Figure 7F). In order to further verify the accuracy of these three genes, ROC curve verification was performed in another dataset (GSE56116). The AUC value of *TUBB* was 0.778 (Figure 7G), that of *CLTA* was 0.778 (Figure 7H), and that of *FBXL5* was 0.889 (Figure 7I). Furthermore, we validated these three genes using clinical samples, and the results showed that the expression of *TUBB* and *CLTA* was significantly higher in the control group than in PMOP. On the contrary, the expression of *FBXL5* in PMOP was significantly higher than that in normal samples (Figure 7J).

### To construct a nomogram model of postmenopausal osteoporosis and premenopausal classification

**Figure 8.**
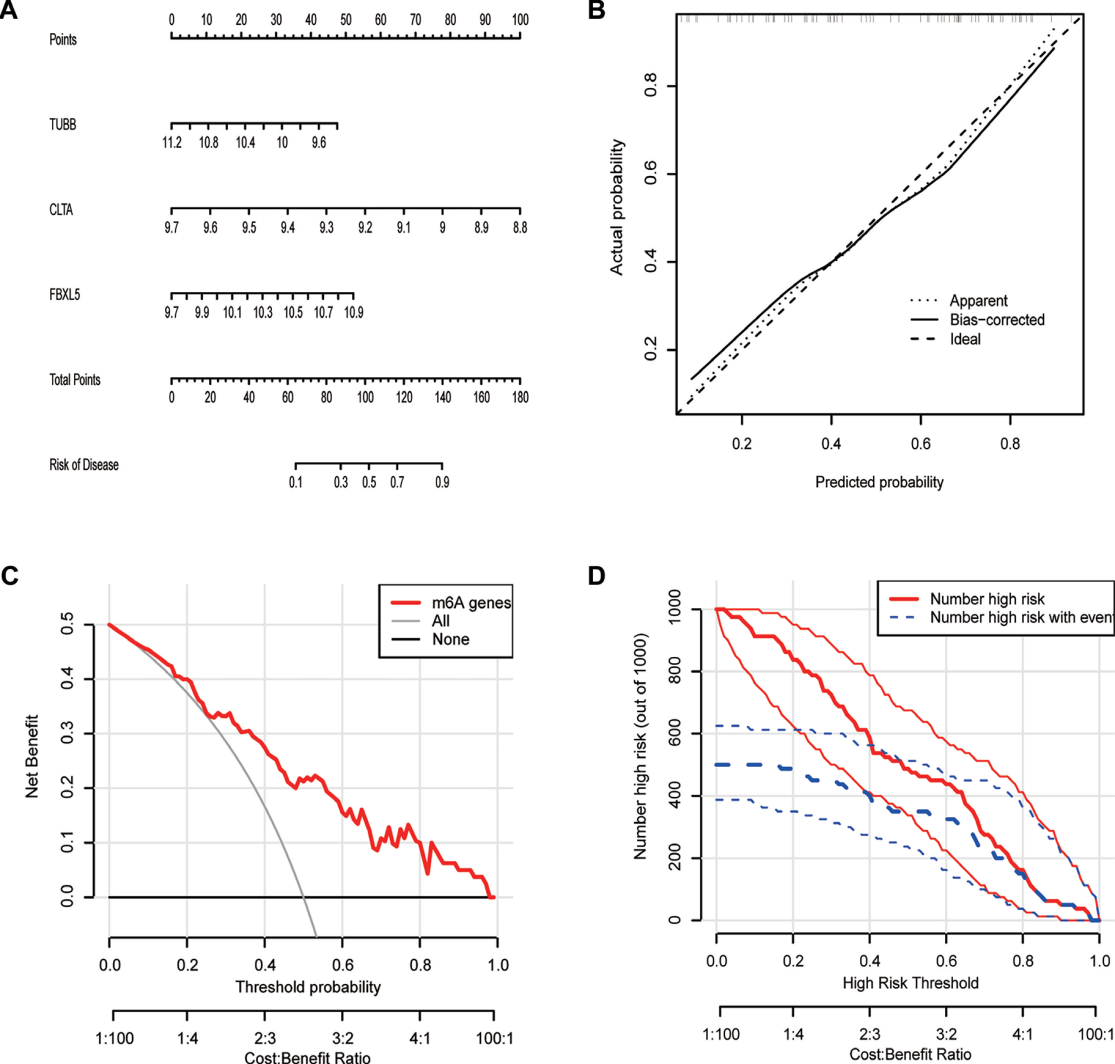
**Developing a postmenopausal and premenopausal nomogram model.** (A) A nomogram model was created considering the selected diagnostic indicators (*TUBB*, *CLTA*, *FBXL5*); (B) A description of the nomogram model’s diagnostic capacity calibration curve; (C) Based on DCA, nomogram models have more clinical usefulness than individual diagnostic indicators; (D) Clinical impact curves reveal that the nomogram model possesses a high capacity for diagnosis.

Using these diagnostic markers, we constructed a nomogram to predict PMOP (Figure 8A). By scoring the above features, the higher the total score, the greater the probability of PMOP occurring. Through the calibration curve, we observed a strong agreement between the predicted and actual probabilities of the nomogram (Figure 8B). DCA revealed that although both the nomogram model and a single diagnostic marker created a net profit, the one from the nomogram model was much higher (Figure 8C). Consequently, nomogram models can be more clinically useful than individual diagnostic indicators. The examination of the clinical influence curve demonstrates that the nomogram model possesses good diagnostic accuracy (Figure 8D).

### Analysis of GSEA

**Figure 9.**
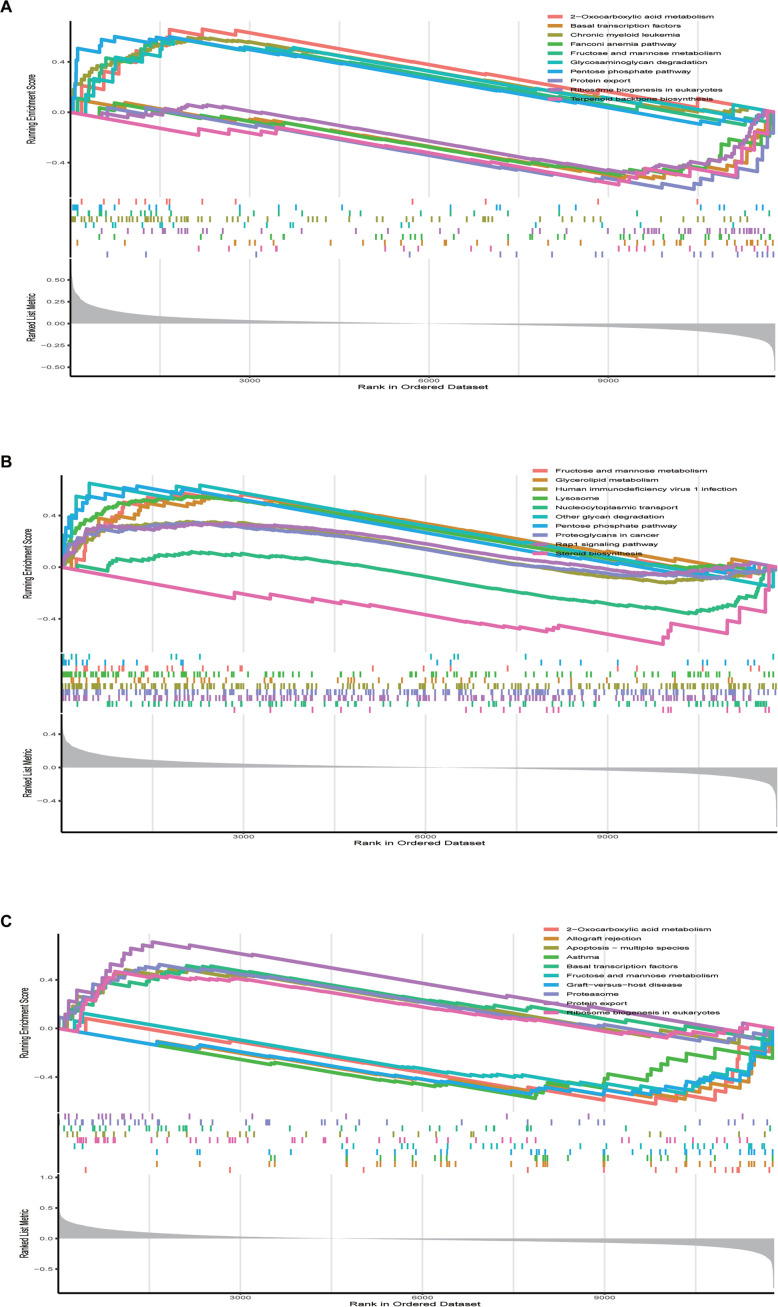
**GSEA analysis Results of diagnostic markers.** (A) GSEA analysis results of *TUBB*; (B) GSEA analysis results of *CLTA*; (C) GSEA analysis results of *FBXL5*.

The GSEA analysis was executed for these three key biomarkers, and the results recorded for *TUBB* showed that high expression of *TUBB* may affect 2-oxocarboxylic acid metabolism and the pentose phosphate pathway. Low expression is affected by protein export as well as pentose and basal transcription factors (Figure 9A).

*CLTA* showed that high expression of *CLTA* may affect the pentose phosphate pathway and fructose and mannose metabolism. Low expression affects steroid biosynthesis and nucleocytoplasmic transport (Figure 9B). The GSEA analysis of *FBXL5* showed that high expression of *FBXL5* may affect protein export, basal transcription factors, and ribosome biogenesis in eukaryotes. Low expression of the gene is affected by fructose and mannose metabolism and 2-oxocarboxylic acid metabolism (Figure 9C).

## Discussion

The prevalence of osteoporosis is particularly high in postmenopausal women of older age, thereby placing these women at risk of fractures. Regarding this age range, the increasing morbidity and mortality linked with hip and spinal fractures have been somewhat worrying. The main purpose of osteoporosis treatment is to stop bones from fracturing through various means such as slowing or blocking bone loss, maintaining bone strength, and reducing or eliminating causes of fractures [19].

In eukaryotes, the major epigenetic modification that performs various functions in biological processes is the N6-methyladenosine (m6A) modification, which is involved in the biological onset and progression of various diseases. Accumulating data suggest that m6A modification plays a dominant role in bone development and osteoporosis metabolism as a novel epigenetic transcriptome marker [20, 21]. Currently, data from many experiments have illustrated the role of m6A modifications in osteoporosis by linking various molecular mechanisms involved in this disease to m6A modifications. Chen et al. [22] found that the osteogenic differentiation of mesenchymal stem cells (MSCs) was enhanced through m6A demethylation mediated by FTO in the 3’ UTR of PPARG mRNA. Peng et al. [23] found that osteogenesis could be promoted through the m6A methylation of LINC00657 mediated by METTL3. Li et al. [24] identified six novel PMOP-related genes using genome-wide association and transcriptome prediction models. The role of bone metabolism, the bone marrow microenvironment, and the immune system is crucial. As such, future research should integrate GWAS, transcriptomics, and m6A methylation data to further investigate the precise role of m6A in the regulation of PMOP-associated genes in order to elucidate a more precise PMOP molecular regulatory network and provide new targets for personalized therapy. Wu et al. [25] showed that the m6A methyltransferase METTL3 in bone marrow MSCs (BMMSCs) could induce osteoporosis in mice, and overexpression of METTL3 protected mice from estrogen deficiency-induced osteoporosis. Therefore, searching for distinct diagnostic markers, analyzing the infiltration pattern of PMOP immune cells, mining the database related to PMOP to find more characteristic genes, providing new ideas for the treatment and prevention of PMOP, and promoting the prognosis of PMOP cases are of great significance.

The three different PMOP subtypes were identified in this research. PMOP cluster B was considerably associated with basic transcription factors and peripheral blood CD56 dim cells, immature dendritic cells, macrophages, and monocytes. In the PMOP C cluster, immature B cells were significantly associated. The association between genes constructing the genetic signatures and immune cells was examined. The analysis indicated a significant association of FMR1 with immature B cells, immature dendritic cells, and macrophages. RBM15 was significantly associated with gamma delta T cells, immature B cells, and CD56 dim natural killer cells. RBM15B was significantly associated with activated CD8 T cells and activated B cells. YTHDC1 is significantly associated with immature B cells. ZC3H13 is significantly associated with mast cells. A large body of literature has studied the relationship between immune cells and bone. Macrophages can affect the formation of osteocytes through paracrine signaling or direct cell-to-cell contact and can also secrete reactive oxygen species (ROS) and inflammatory cytokines (IL-1β, IL-6, TNF-α) to influence the formation of osteoclasts [26]. Mast cells themselves contain many osteoclast mediators, including IL-6 and TNF-α. Kroner et al. showed that stimulated mast cell supernatant induced osteoclast genesis when estrogen was absent [27, 28].

Several experiments have indicated that the lack of estrogen is an important factor that affects the formation of postmenopausal osteoporosis. In a physiological state, estrogen utilizes various signaling pathways to protect osteoblasts from apoptosis, enhance the proliferation, maturation, and ossification of osteoblasts, and maintain the formation of bone [29, 30]. The decrease in estrogen levels in postmenopausal women eliminates this protective effect on bone, promotes osteoclast generation and bone resorption, and inhibits apoptosis of osteoclasts through various processes [29, 31]. Manolagas et al. pointed out that the loss of estrogen reduces the defense against oxidative stress in bone and unbalances the REDOX reaction. This excessive increase in ROS leads to the excessive proliferation of osteoclasts, resulting in a gradual decline in the volume and density of bone tissue [32]. The role of estrogen in the formation of osteoporosis through its effect on the immune system has been well established in many studies. Estrogen can inhibit osteoclast formation by down-regulating T lymphocytes and other inflammatory cytokines. Estrogen deficiency increases T cell activity, upregulates some inflammatory cytokines (IL-1β, IL-6, TNF-α), increases the expression of NF-κB Ligand (RANKL), and stimulates osteoclast production and bone resorption [33, 34]. Under conditions of estrogen deficiency, B lymphocytes regulate the production of osteoclasts by secreting RANKL, while neutrophils become overactivated, leading to osteoblast apoptosis and increased osteoclast production by releasing ROS [28]. Because our knowledge of the relationship between the immune system, estrogen, and osteoporosis is incomplete, further study is required. A potentially beneficial strategy to treat PMOP would be to target the immune system. In addition, the intersection genes of these three different PMOP isoforms were analyzed by GO enrichment analysis, and the results indicated a significant association of the 302 PMOP central genes with biological processes such as macrophage autophagy and protein dephosphorylation. Osteoporosis is characterized by reduced bone mass and significant adipose tissue accumulation within the bone marrow environment. Autophagy is extremely important in the elimination of dysfunctional or unnecessary organelles and proteins [35].

Recent research has shown that m6A modification plays a vital role in the regulation of autophagy and adipogenesis [36]. Qi et al. [37] found that autophagy affects the development of PMOP by regulating endogenous BMMSCs. Jiao et al. [38] found that m6A modification could inhibit adipocyte differentiation. Singh et al. [39] found that autophagy regulates fat accumulation and lipogenesis. Recent studies have shown that dephosphorylation not only plays a crucial role in the control of osteogenesis and adipogenesis [40] but is also involved in the regulation of chondrocytes [41]. Aging or other pathological stimuli affect the imbalance of protein phosphorylation, which contributes to bone marrow obesity and progressive bone loss, leading to the development of osteoporosis [40].

In this study, LASSO, RF, and SVM-RFE algorithms were performed using previously obtained 302 central genes. Three hub genes related to PMOP were identified from the intersection of genes obtained by these three algorithms. Later, to identify the three characteristic genes, *CLTA*, *TUBB*, and *FBXL5*, the ROC curve was used for analysis, and the results indicated that the gene marker under study had excellent predictive ability. At present, no relevant studies have investigated the mechanism between CLTA and PMOP. Huang et al. found that *CLTA* was linked with rheumatoid arthritis [42]. They discovered that CCAAT/enhancer binding protein β (C/EPPβ) combined with *CLTA* was involved in FAO metabolism, which caused osteoclast overgeneration and bone destruction. At the same time, many studies have confirmed that the FAO metabolic mechanism is involved in osteoclast metabolism [43, 44], and it can be assumed that *CLTA* may also affect PMOP by similarly causing bone loss. The mechanism of this interaction needs to be further studied. *TUBB* is one of the components that form microtubules. Previous studies have shown that microtubules affect bone resorption through their effects on the actin cytoskeleton of osteoclasts [45, 46]. The relationship between microtubules and the actin cytoskeleton is physiological and functional [46]. The actin cytoskeleton and microtubules are required for the development of foot processes and osteoclast bone resorption [47]. Kodama [48] pointed out that microtubules mediate the formation of podosomes by microfilaments and affect the differentiation and maturation of osteoclasts.

At present, the mechanism of action between *TUBB* and PMOP is not yet known, although it is believed that *TUBB* may also affect the generation of osteoclasts by regulating osteoclast activity, thus leading to OP. Iron is an important substance in human cell metabolism. Numerous investigations conducted recently have revealed a clear association between abnormal iron metabolism and osteoporosis [49]. Postmenopausal women experience an accumulation of iron along with estrogen deficiency. According to Chen et al., increased iron levels are a risk factor for PMOP in postmenopausal women [50]. A disorder of iron metabolism affects bone homeostasis [51]. In addition to promoting osteoclast differentiation and osteoblast death, excess iron decreases osteoblast proliferation and differentiation [50]. Several studies have indicated a close correlation of *FBXL5* with iron metabolism, and *FBXL5* can affect cellular iron metabolism by mediating iron regulatory protein 1 (IRP1) and iron regulatory protein 2 (IRP2) [51, 52]. According to Liu et al., iron buildup controls osteoblast apoptosis through the lncRNA XIST/Mir-758-3p/caspase 3 axis, leading to osteoporosis [53]. Although the direct mechanism of *FBXL5* and PMOP has not been studied, we believe that *FBXL5* is likely to cause osteoporosis by affecting iron metabolism.

Despite this, there are still some limitations in this study, which systematically analyzed the GEO public database and confirmed that *TUBB*, *CLTA,* and *FBXL5* could be identified as potential diagnostic markers of PMOP using ML algorithms. First of all, this study is mostly based on bioinformatics data mining, and further experimental validation of the biological functions of these markers in PMOP progression is warranted. Second, the heterogeneity of the samples may affect the universality of the analysis results because the data are obtained from public databases. Therefore, to further prove the reliability and application value of this study, future studies should confirm the specific mechanisms of *TUBB*, *CLTA,* and *FBXL5* in PMOP development via cell experiments, animal models, and clinical samples, and evaluate their utility value with respect to clinical diagnosis and treatment.

## Conclusion

This study has identified and validated that *TUBB*, *CLTA,* and *FBXL5* could serve as potential diagnostic markers for PMOP and has shown their utility in early screening, personalized risk assessment, and targeted therapy. These genes can serve as non-invasive blood tests to increase the early diagnosis rate of PMOP and be potential therapeutic targets for iron metabolism regulation, microtubule stability, and immune intervention. Future work should integrate clinical validation, multi-omics analysis, and novel molecular intervention strategies to facilitate the precise diagnosis and treatment of PMOP and offer patients more effective prevention and treatment measures.
